# The relationship between dietary total flavonoids and thyroid function in U.S.adults, NHANES 2007–2010

**DOI:** 10.1371/journal.pone.0303169

**Published:** 2024-05-21

**Authors:** Jie Wu, Chuyu Jia, Zirui Zhang, Zebin Hou, Yanhua Cui

**Affiliations:** 1 Department of Thyroid Surgery, Shanxi Provincial People’s Hospital, Taiyuan, Shanxi, China; 2 Department of Physical Examination Center, Shanxi Provincial People’s Hospital, Taiyuan, Shanxi, China; 3 Fifth Clinical Medical College, Shanxi Medical University, Taiyuan, Shanxi, China; 4 Department of Ultrasound, Shanxi Provincial People’s Hospital, Taiyuan, Shanxi, China; New York University Grossman School of Medicine, UNITED STATES

## Abstract

**Background:**

Although small studies have shown that flavonoids can affect thyroid disease, few epidemiological studies have explored the relationship between dietary total flavonoids (TFs) intake and serum thyroid function. The aim of this research was to evaluate the relationship between TFs and serum thyroid function.

**Methods:**

Our study included 4,949 adults from the National Health and Nutrition Examination Survey (NHANES) 2007–2010. Multivariable linear regression, subgroup analyses, and interaction terms were used to explore the relationships between TFs and thyroid function. And we also used restricted cubic splines (RCS) to investigate possible nonlinear relationships.

**Results:**

After adjusting for covariates, we found that log10-transformated dietary total flavonoids intake (LgTFs) was negatively associated with total thyroxine (TT4) (β = -0.153, 95% CI = -0.222 to -0.084, *P*<0.001). Subgroup analyses revealed a stronger and statistically supported association in subjects with high annual family income (β = -0.367, *P*<0.001, *P* for interaction = 0.026) and subjects with high poverty to income ratio (PIR) (β = -0.622, *P*<0.001, *P* for interaction = 0.042). And we found a U-shaped curve association between LgTFs and free triiodothyronine (FT3) (inflection point for LgTFs: 2.063).

**Conclusion:**

The results of our study demonstrated that a higher intake of total flavonoids in the diet was negatively associated with a lower TT4. Furthermore, the associations were more pronounced in high annual family income and high PIR adults. And we found a U-shaped relationship between LgTFs and FT3. These findings provided guidance for future thyroid dysfunction diet guidelines.

## Introduction

Thyroid hormone is secreted mainly by the thyroid, which can promote metabolic function, growth, reproduction, and neurological development [1]. Thyroid dysfunction is common, readily recognized, and easily treatable, and its incidence has increased significantly in recent decades [2]. Previous studies showed that the prevalence of hypothyroidism to be 11.7% and the prevalence of hyperthyroidism to be 2.5% in the U.S. population [3,4]. Many other risk factors, including genetic susceptibility [5], diet [6], smoking status [7], sex [8] and drugs [9], are known to interfere with thyroid hormone metabolism and biosynthesis. Therefore, it is very important to explore the factors affecting thyroid function for the prevention and treatment of thyroid diseases.

Flavonoids are a large group of secondary metabolites that are widely found in fruits and vegetables, as well as nuts, seeds, coffee, wine, dark tea, and green tea [10,11]. Flavonoids have a variety of physiological activities and functions, including resistance inflammation, bacteriostatic, antioxidant, anti-tumor,anti-cancer and other characteristics [12]. These biological activities play an important role in keeping human health and have been widely used in food, medicine, and other fields [13].

Although the role of flavonoids in human health was not fully clear, recent studies have shown that flavonoids can affect thyroid function through multiple mechanisms [14–16]. However, few epidemiological studies have explored the associations of flavonoids with measures of thyroid function. Therefore, the objective of our investigation was to evaluate the relationship between dietary total flavonoids (TFs) intake and serum thyroid function in adults in the United States (US).

## Methods

### Study population

Data were extracted from the National Health and Nutrition Examination Survey (NHANES), which is a nationally cross-sectional survey to assess the health and nutrition status of the US population. The Research Ethics Review Board of the US National Center for Health Statistics (NCHS) approved the NHANES protocol, and all participants signed the informed consent before the start of the investigation. Publicly available data sets were analyzed in this study. All data are available from the NHANES (https://www.cdc.gov/nchs/nhanes/index.htm).

**Fig 1:**
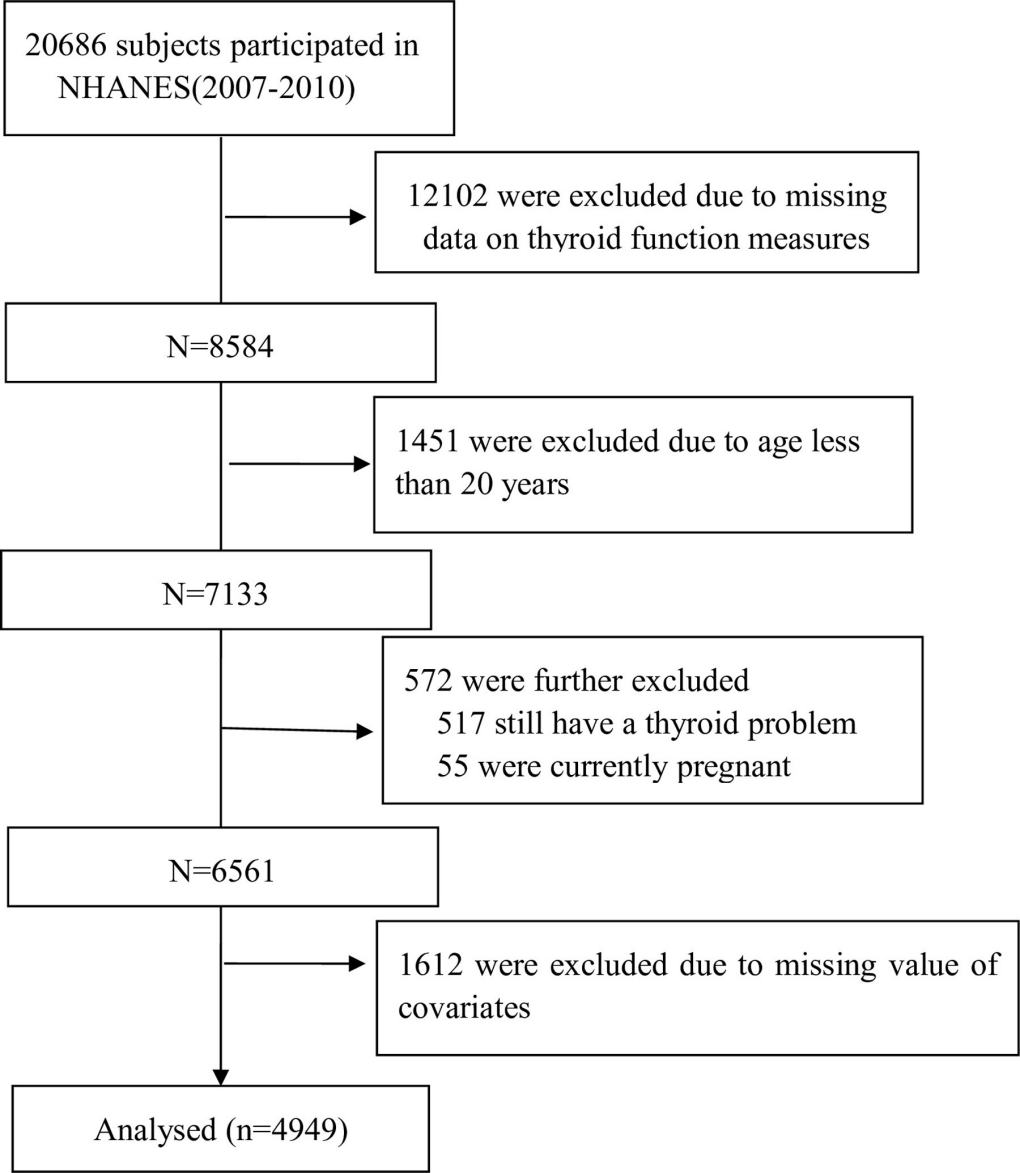
Flow diagram.

A total of 20,686 participants from 2007 to 2010 NHANES data for present study. We excluded participants who missed thyroid measure data (N = 12,102), who aged less than 20 years (N = 1,451) or still have a thyroid problem (N = 517) or pregnant now (N = 55). Also, with the missing value data (N = 1,612). Finally, 4,949 subjects included in our analysis (Fig 1).

### Serum thyroid measure

The thyroid panel are consists of a battery of several tests for the measurement of thyroid function, including total thyroxine (TT4), total triiodothyronine (TT3), free thyroxine (FT4), free triiodothyronine (FT3) and thyroid stimulating hormone (TSH). Detailed measurement methods of thyroid blood specimens collecting and processing are provided in the NHANES Laboratory Procedure Manual. We analyzed these measurements from the 2007 to 2010 NHANES cycles.

### Total flavonoids intakes measurement

The development and calculation of the intake of total dietary flavonoids have been previously reported [17], the TFs was estimated using the average of two 24h dietary recall data of the NHANES. The dietary two-day sample weight (WTDR2D) was applied for weighting. Moreover, for the non-normal distribution of TFs data, we used logarithmic transformations to obtain normal distributions of data (named LgTFs) by validation of Shapiro-Wilks tests. The LgTFs were categorized into quartiles as follows: Q1 = 0.031 to 1.399; Q2 = 1.400 to 1.791; Q3 = 1.792 to 2.364; Q4 = 2.365 to 3.844.

### Covariates

Based on previous studies and clinical consideration, we selected potential covariates that could play a role in the associations between TFs and thyroid function. Demographic information was collected through standardized household interviews, including age, sex, education level, race/ethnicity, annual family income, poverty to income ratio (PIR), body mass index (BMI), alcohol consumption, and smoking status.

Age was divided into three age groups (20–40, 41–60 and >60 years). Race/ethnicity was divided into non-Hispanic white, non-Hispanic black, Hispanic and other race. The education level was graded as less than high school, high school graduate, and more than high school. Three categories of annual family income were: < 25,000, 25,000 to 74,999 and ≥75,000$. PIR was categorized as: <1.0; 1.0–1.9; 2.0–3.9; ≥4.0. BMI was categorized as: <25.0, 25.0–29.9, and ≥30 kg/m2. We divided alcohol consumption into three groups: ≤50, 51 to ≤100 and >100 g/day. Smoking status was classified as never (smoked less than 100 cigarettes in life), former (smoked more than 100 cigarettes in life and smoke not at all now), or now (smoked more than 100 cigarettes in life and smoke some days or every day).

### Statistical analysis

All statistical analyses were performed in package R (Version 4.2.2, R Foundation for Statistical Computing, Vienna, Austria). Continuous variables are displayed as mean with standard deviation (SD), and categorical variables are expressed as frequency with percentage. The Chi-square test for categorical variables and one-way analysis of variance (ANOVA) for continuous variables to assess total flavonoids intake by social demographic variables.

Multivariable linear regression models were used to explore the association between total flavonoids intake and measures of thyroid function. Model 1 did not adjust for any variable. Model 2 adjusted for age, gender, education level, race/ethnicity, annual family income, PIR, BMI, alcohol consumption and smoking status. The results were further used with restricted cubic splines (RCS) to explore possible nonlinear relationships.

Finally, subgroup analyses and interaction tests were performed for each covariate to evaluate the heterogeneity of the associations between LgTFs and total thyroxine (TT4). Statistical significance was considered as P-value <0.05. In this study, we used the WTDR2D as all data weights.

## Results

### Baseline participant characteristics

The distributions of demographic, socioeconomic and dietary characteristics by TFs quartiles were presented in **Table 1**. A total of 4,949 adults from the NHANES data for 2007 to 2010 were incorporated into the present analysis, among which 2,422 (50.680%) were females and 2,527 (49.320%) were males. The consumption of TFs among these participants was 228.945 (9.757) mg/day. In the field of age, race, annual family income, PIR, smoking status, alcohol use, BMI, and level of education, there was a statistically difference among LgTFs quartiles(all *P*<0.05). For thyroid parameters, the study revealed significant differences in FT3, TT3, TT4, and FT3/TT4 ratio, which among all participants were 3.187(0.011) pg/ml, 1.782 (0.018) nmol/L, 7.760 (0.050) ug/dl and 1.317 (0.009), respectively.

**Table 1:** Baseline characteristics of the NHANES (2007–2010) study population in LgTFs quartiles.

Characreristics	LgTFs quartiles					P-value
	Overall	Q1	Q2	Q3	Q4	
N(%)	4949(100)	1237(24.758)	1237(23.592)	1237(22.644)	1238(29.006)	
LgTFs	[0.031,3.844]	[0.031,1.399]	(1.399,1.791]	(1.791,2.364]	(2.364,3.844]	
LgTFs	1.898(0.023)^**a**^	1.081(0.014)	1.598(0.004)	2.045(0.008)	2.723(0.011)	**<0.0001**
TFs	228.945(9.757)	12.821 (0.333)	39.987 (0.367)	118.574 (2.539)	653.283(20.278)	**<0.0001**
**Age(years)**	45.879(0.462)	42.994(0.810)	45.667(0.760)	48.145(0.791)	46.746(0.496)	**<0.0001**
**Gender**						0.095
Female	2422(50.680)^**b**^	634(53.778)	585(46.331)	598(49.543)	605(52.462)	
Male	2527(49.320)	603(46.222)	652(53.669)	639(50.457)	633(47.538)	
**Race**						**0.003**
White	2459(70.423)	609(70.140)	574(67.547)	548(66.756)	728(75.866)	
Black	953(10.662)	265(12.374)	230(10.853)	238(11.057)	220 (8.737)	
Hispanic	836 (8.510)	194 (8.246)	258(10.867)	249(10.988)	135 (4.882)	
other race	701(10.405)	169 (9.239)	175(10.733)	202(11.199)	155(10.515)	
**Family income($)**						**<0.0001**
<25,000	1692(26.259)	509(33.877)	439(27.065)	398(22.896)	346(21.727)	
25,000–74,999	2157(42.471)	546(44.700)	535(39.465)	545(41.877)	531(43.478)	
> = 75,000	1100(31.270)	182(21.423)	263(33.470)	294(35.227)	361(34.795)	
**Education**						**<0.0001**
<high school	1416(19.588)	446(27.667)	377(20.483)	349(18.300)	244(12.970)	
High School	1189(24.158)	323(26.944)	295(25.071)	264(20.135)	307(24.177)	
> high school	2344(56.254)	468(45.389)	565(54.446)	624(61.565)	687(62.853)	
**PIR**						**<0.0001**
<1.0	1004(14.571)	318(19.786)	274(16.112)	236(12.456)	176(10.517)	
1.0–1.9	1348(20.732)	397(27.010)	325(18.555)	319(18.633)	307(18.784)	
2.0–3.9	1336(27.783)	333(29.022)	332(27.188)	353(30.028)	318(25.458)	
> = 4.0	1261(36.914)	189(24.182)	306(38.145)	329(38.883)	437(45.241)	
**BMI (kg/m** ^**2**^ **)**						**0.022**
<25	1390(31.441)	328(28.939)	359(32.617)	363(33.213)	340(31.237)	
25–29.9	1726(33.708)	382(29.397)	432(34.332)	470(35.194)	442(35.718)	
= >30	1833(34.851)	527(41.664)	446(33.051)	404(31.592)	456(33.045)	
**Alcohol (g/d)**						**< 0.001**
< = 50	4735(94.806)	1213(97.678)	1160(91.017)	1173(94.219)	1189(95.895)	
51–100	175 (4.239)	22(2.165)	64(6.983)	50(4.674)	39(3.438)	
>100	39 (0.955)	2(0.157)	13(2.000)	14(1.106)	10(0.667)	
**Smoke**						**<0.0001**
never	2580(52.997)	552(44.914)	673(56.092)	703(58.713)	652(52.917)	
former	1310(24.926)	296(21.711)	317(23.455)	356(26.738)	341(27.452)	
now	1059(22.077)	389(33.375)	247(20.453)	178(14.549)	245(19.631)	
**FT4 (pmol/L)**	10.009(0.077)	10.095(0.132)	10.055(0.114)	9.932(0.082)	9.959(0.094)	0.559
**FT3 (pg/mL)**	3.187(0.011)	3.237(0.022)	3.190(0.015)	3.157(0.013)	3.167(0.018)	**0.013**
**TSH(uIU/mL)**	1.978(0.048)	1.934(0.099)	1.990(0.101)	1.940(0.059)	2.034(0.054)	0.51
**TT3(nmol/L)**	1.782(0.018)	1.834(0.020)	1.786(0.024)	1.749(0.023)	1.761(0.022)	**0.007**
**TT4(ng/mL)**	7.760(0.050)	7.958(0.082)	7.770(0.058)	7.705(0.069)	7.625(0.070)	**< 0.001**
**FT3/TT3**	1.906(0.023)	1.876(0.022)	1.893(0.027)	1.929(0.036)	1.926(0.027)	0.147
**FT4/TT4**	1.317(0.009)	1.295(0.015)	1.322(0.013)	1.317(0.013)	1.331(0.011)	**0.042**
**FT3/fT4**	0.327(0.002)	0.330(0.004)	0.325(0.004)	0.326(0.003)	0.325(0.002)	0.514
**TT3/TT4**	0.235(0.003)	0.236(0.003)	0.235(0.004)	0.233(0.003)	0.235(0.004)	0.886

Notes: a Continuous variable: weighted mean (SD). b Categorical variables: actual frequencies (weighted percentages). Abbreviations: SD, standard deviation; TFs, total flavonoids; LgTFs, Logarithm-transformed total flavonoids; PIR, poverty to income ratio; BMI, body mass index; TT4, total thyroxine; TT3, total triiodothyronine; FT4, free thyroxine; FT3, free triiodothyronine; TSH, thyroid stimulating hormone.

### The relationship between LgTFs and thyroid function

The relationship between LgTFs and thyroid function was presented in **Table 2**. In the unadjusted model 1, LgTFs negatively associated with TT4 (β = -0.188, 95% CI = -0.266 to -0.110, *P*<0.0001), after adjustment for covariates in model 2 the associations remained (β = -0.153, 95% CI = -0.222 to -0.084, *P*<0.001). This association was also presented after categorizing LgTFs into quartiles (*P* for trend = 0.001), and participants in the highest LgTFs quartiles (Q4) had a lower TT4 than those in the lowest LgTFs quartiles (Q1) (β = -0.268, 95% CI = -0.409 to -0.127, *P* = 0.002) in model 2.

**Table 2:** The associations between LgTFs and thyroid function.

	TT3(nmol/L)	TT4(ng/mL)	FT3 (pg/mL)	FT4/TT4
**Model 1 β (95%CI) P value**				
LgTFs	-0.044(-0.069,-0.018) **0.001**	-0.188(-0.266,-0.110) **<0.0001**	-0.043(-0.074,-0.012) **0.008**	0.018(0.000,0.035) **0.039**
LgTFs quartiles				
Q1	ref	ref	ref	ref
Q2	-0.048(-0.094,-0.003) **0.038**	-0.188(-0.363,-0.013) **0.036**	-0.047(-0.100, 0.006) 0.079	0.026(-0.001,0.054) 0.059
Q3	-0.085(-0.138,-0.032) **0.003**	-0.253(-0.446,-0.059) **0.012**	-0.079(-0.130,-0.029) **0.003**	0.022(-0.021,0.065) 0.299
Q4	-0.073(-0.117,-0.029) **0.002**	-0.333(-0.471,-0.196) **<0.0001**	-0.07(-0.126,-0.013) **0.018**	0.036 (0.008,0.064) **0.015**
p for trend	**0.002**	**<0.0001**	**0.009**	**0.033**
**Model 2 β (95%CI) P value**				
LgTFs	-0.021(-0.047, 0.006) 0.111	-0.153(-0.222,-0.084) **<0.001**	-0.013(-0.040, 0.015) 0.334	0.013(-0.003, 0.030) 0.109
LgTFs quartiles				
Q1	ref	ref	ref	ref
Q2	-0.013(-0.052, 0.026) 0.478	-0.119(-0.312, 0.074) 0.198	-0.023(-0.069, 0.024) 0.302	0.013(-0.018, 0.044) 0.38
Q3	-0.036(-0.086, 0.014) 0.138	-0.219(-0.423,-0.014) **0.039**	-0.027(-0.069, 0.015) 0.183	0.01(-0.033, 0.053) 0.606
Q4	-0.032(-0.076, 0.013) 0.141	-0.268(-0.409,-0.127) **0.002**	-0.02(-0.072, 0.032) 0.407	0.026(-0.003, 0.055) 0.07
p for trend	0.154	**0.001**	0.419	0.089

Notes: The LgTFs was converted from a continuous variable to a categorical variable. Data are presented as OR (95% CI). Model 1 was adjusted no covariates; Model 2 was adjusted for age, gender, education level, race/ethnicity, annual family income, PIR, BMI, alcohol consumption and smoking status. Abbreviations: PIR, poverty index ratio; BMI, body mass index; FT3, free triiodothyronine; TT4, total thyroxine; TT3, total triiodothyronine; LgTFs, Logarithm-transformed total flavonoids; OR, odds ratio; CI, confidence interval; Ref, reference.

LgTFs negatively correlated with TT3 and FT3 in the unadjusted model 1(TT3: β = -0.044, 95% CI = -0.069 to -0.018, *P* = 0.001; FT3: β = -0.043, 95%CI = -0.074 to -0.012, *P* = 0.008). When LgTFs was used as a categorical variable, this negative association was still present (TT3: *P* for trend = 0.002; FT3: *P* for trend = 0.009), but this association did not remain after adjusting for confounders in model 2. In contrast, LgTFs was positively associated with the FT4/TT4 ratio in model 1 (β = 0.018, 95% CI: 0.000 to 0.035, *P* = 0.039). While, after adjusting for covariates this correlation became insignificant.

**Fig 2:**
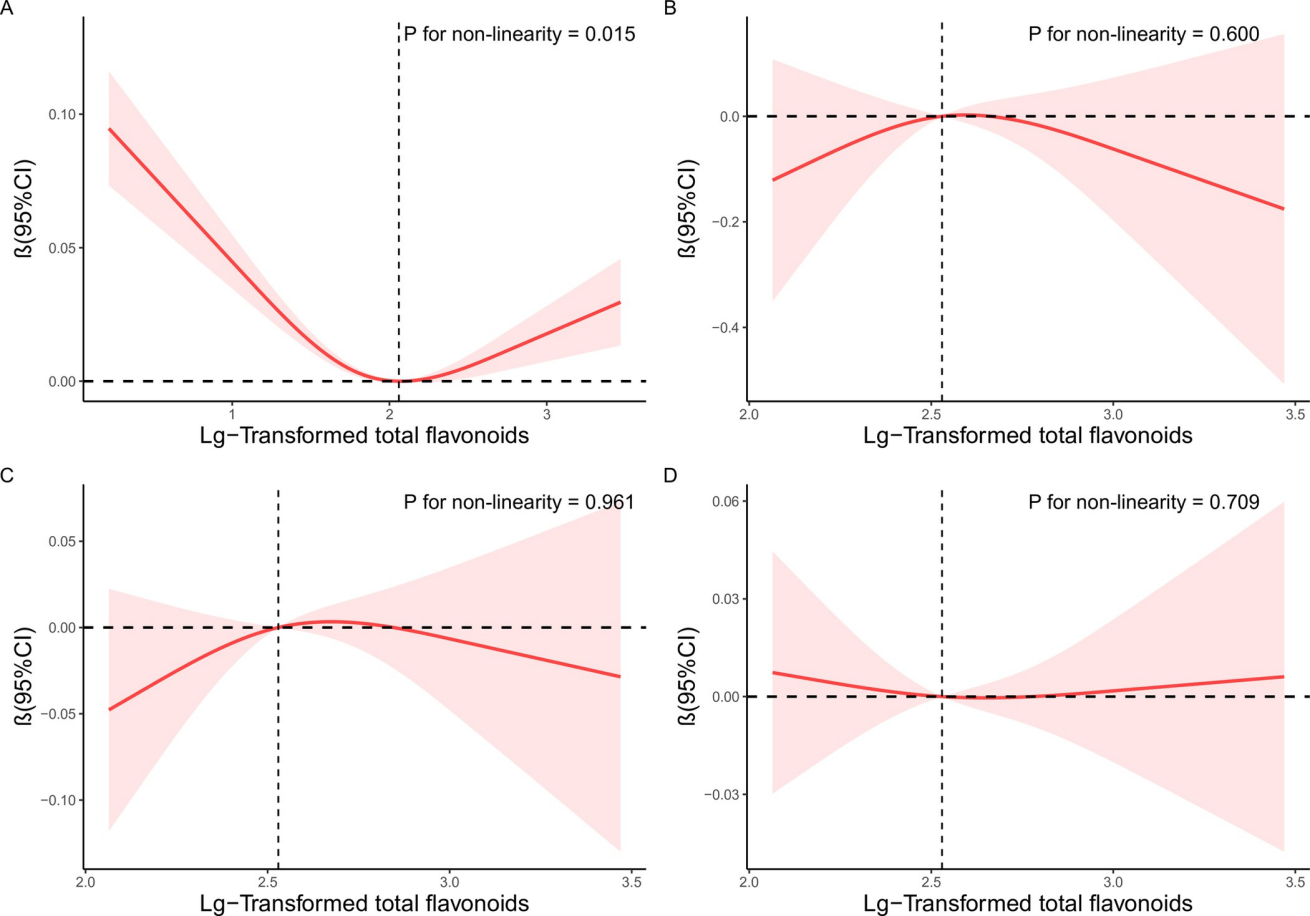
RCS analysis of nonlinear association between FT3, TT4, TT3, FT4/TT4 and LgTFs. Notes: Models adjust for age, gender, education level, race/ethnicity, annual family income, PIR, BMI, alcohol consumption and smoking status. Vertical reference bars identified these inflection points. The red line and the shaded area symbolize the OR and corresponding 95% CI, respectively. (A) RCS curve of the association between logTFs and FT3; (B) RCS curve of the association between logTFs and TT4; (C) RCS curve of the association between logTFs and TT3; (D) RCS curve of the association between logTFs and FT4/TT4. Abbreviations: RCS, restricted cubic spline; PIR, poverty index ratio; BMI, body mass index; FT3, free triiodothyronine; TT4, total thyroxine; TT3, total triiodothyronine; LgTFs, Logarithm-transformed total flavonoids; OR, odds ratio; CI, confidence interval.

In addition, the restricted cubic splines were adopted to explore the nonlinear association between LgTFs and FT3, TT4, TT3, FT4/TT4. After adjusting for possible covariates, a curve relationship U-shaped (*P* for nonlinear = 0.015) was observed between LgTFs and FT3 (**Fig 2A**). We did not find a nonlinear relationship between TT4, TT3, FT4/TT4 and LgTFs (**Fig 2B–2D**). As shown in **Table 3**, the two-step recursive method showed an inflection point at 2.063 for LgTFs.

**Table 3:** Analysis of LgTFs and FT3 using segmented linear regression.

Models	β (95%CI)	P value
**Model I**		
One line effect	-0.013(-0.040, 0.015)	0.334
**Model II**		
Inflection point(K)	2.063	
LgTFs < 2.063	-0.099(-0.176, -0.022)	0.013
LgTFs > 2.063	0.021(-0.063, 0.105)	0.619
P for log-likelihood ratio test		0.002

Notes: Model I, one-line linear regression; Model II, two-segment regression. β was the effect size and the 95% CI indicated the confidence interval. Adjust for: age, gender, education level, race/ethnicity, annual family income, PIR, BMI, alcohol consumption and smoking status. Abbreviations: PIR, poverty index ratio; BMI, body mass index; FT3, free triiodothyronine; TT4, total thyroxine; TT3, total triiodothyronine; LgTFs, Logarithm-transformed total flavonoids; OR, odds ratio; CI, confidence interval.

### Stratified analysis and interaction tests of correlations between LgTFs and TT4

Our study demonstrated that LgTFs was negatively associated with TT4, so we further evaluated the effect of LgTFs on TT4 in different subgroups. The detailed findings of the stratified analysis was shown in **Table 4**. When LgTFs was used as a categorical variable, PIR had a significant interaction (*P* for interaction = 0.042). High PIR groups (PIR >4) had a greater decrease in TT4 levels compared to other groups (β = -0.622, 95% CI: -0.940 to -0.305, *P*<0.001). When LgTFs was used as a continuous variable, annual family income played an interactive role in the relationship between LgTFs and TT4. And this association was more significant in individuals with a high annual family income individuals (β = -0.367, 95% CI: -0.523 to -0.210, *P*<0.001, *P* for interaction = 0.026).

**Table 4:** Relationship between LgTFs and TT4 in each subgroup.

	LgTFs			LgTFs quartiles					
Characreristics	95% CI	p	p for interaction	Q1	Q2	Q3	Q4	p for trend	p for interaction
**Age(years)**			0.632						0.494
20–40	-0.14(-0.245,-0.034)	**0.013**		ref	-0.091(-0.354, 0.173)	-0.271(-0.600, 0.059)	-0.274(-0.498,-0.050)	**0.014**	
41–60	-0.177(-0.302,-0.051)	**0.009**		ref	-0.154(-0.482, 0.174)	-0.298(-0.602, 0.006)	-0.286(-0.507,-0.065)	**0.023**	
>60	-0.08(-0.277, 0.116)	0.396		ref	-0.08(-0.470, 0.309)	0.102(-0.281, 0.486)	-0.133(-0.480, 0.214)	0.606	
**Gender**			0.237						0.666
Female	-0.093(-0.252, 0.067)	0.231		ref	-0.089(-0.419, 0.241)	-0.151(-0.471, 0.169)	-0.211(-0.509, 0.087)	0.146	
Male	-0.208(-0.370,-0.046)	**0.016**		ref	-0.164(-0.401, 0.074)	-0.281(-0.551,-0.011)	-0.319(-0.584,-0.054)	**0.014**	
**Race**			0.572						0.62
White	-0.166(-0.248,-0.084)	**<0.001**		ref	-0.11(-0.347, 0.127)	-0.209(-0.476, 0.059)	-0.273(-0.433,-0.113)	**0.001**	
Black	-0.115(-0.379, 0.150)	0.357		ref	-0.061(-0.676, 0.554)	-0.151(-0.719, 0.417)	-0.342(-0.819, 0.134)	0.124	
Hispanic	-0.032(-0.263, 0.199)	0.762		ref	-0.287(-0.629, 0.054)	-0.34(-0.652,-0.028)	0.056(-0.314, 0.427)	0.839	
other race	-0.197(-0.478, 0.084)	0.152		ref	-0.011(-0.442, 0.420)	-0.166(-0.640, 0.308)	-0.344(-0.859, 0.170)	0.116	
**Family income**			**0.026**						0.124
<25,000	-0.035(-0.186, 0.116)	0.625		ref	-0.073(-0.397, 0.251)	-0.12(-0.475, 0.236)	-0.018(-0.293, 0.257)	0.797	
25,000–74,999	-0.088(-0.237, 0.060)	0.223		ref	-0.15(-0.377, 0.077)	-0.09(-0.302, 0.121)	-0.179(-0.438, 0.080)	0.21	
> = 75,000	-0.367(-0.523,-0.210)	**<0.001**		ref	-0.233(-0.818, 0.353)	-0.532(-0.975,-0.089)	-0.661(-1.069,-0.254)	**<0.001**	
**Education**			0.971						0.809
<high school	-0.135(-0.305, 0.036)	0.113		ref	-0.157(-0.481, 0.168)	-0.183(-0.552, 0.186)	-0.182(-0.453, 0.088)	0.178	
High School	-0.172(-0.379, 0.036)	0.098		ref	-0.179(-0.497, 0.139)	-0.293(-0.655, 0.068)	-0.24(-0.617, 0.137)	0.176	
> high school	-0.143(-0.248,-0.039)	**0.011**		ref	-0.049(-0.409, 0.311)	-0.158(-0.495, 0.178)	-0.288(-0.486,-0.090)	**0.001**	
**PIR**			0.093						**0.042**
<1.0	-0.099(-0.392,0.195)	0.486		ref	-0.24(-0.683, 0.202)	-0.462(-0.906,-0.018)	-0.017(-0.485, 0.450)	0.624	
1.0–1.9	0.028(-0.114, 0.169)	0.683		ref	0.106(-0.205, 0.417)	-0.022(-0.372, 0.329)	0.053(-0.205, 0.310)	0.86	
2.0–3.9	-0.197(-0.365,-0.029)	**0.025**		ref	-0.094(-0.397, 0.208)	-0.041(-0.423, 0.340)	-0.295(-0.603, 0.014)	0.069	
> = 4.0	-0.279(-0.439,-0.119)	**0.002**		ref	-0.348(-0.815, 0.119)	-0.493(-0.815,-0.172)	-0.622(-0.940,-0.305)	**<0.001**	
**BMI (kg/m** ^**2**^ **)**			0.185						0.46
<25	-0.265(-0.390,-0.141)	**<0.001**		ref	-0.078(-0.435, 0.280)	-0.212(-0.526, 0.103)	-0.394(-0.681,-0.107)	**0.002**	
25–29.9	-0.071(-0.230, 0.088)	0.351		ref	-0.151(-0.528, 0.225)	-0.093(-0.490, 0.303)	-0.209(-0.561, 0.144)	0.243	
= >30	-0.129(-0.248,-0.010)	**0.036**		ref	-0.14(-0.373, 0.094)	-0.336(-0.582,-0.091)	-0.206(-0.431, 0.018)	**0.036**	
**Alcohol (g/d)**			0.198						0.07
< = 50	-0.14(-0.209,-0.071)	**<0.001**		ref	-0.112(-0.297, 0.072)	-0.184(-0.394, 0.026)	-0.248(-0.385,-0.110)	**<0.001**	
51–100	-0.502(-1.137,0.133)	0.104		ref	-0.831(-1.848, 0.186)	-1.274(-2.450,-0.098)	-1.089(-2.386, 0.207)	0.111	
>100	0.574(-0.084, 1.231)	0.073		ref	0.012(-2.388, 2.411)	0.212(-2.214, 2.638)	0.959(-0.761, 2.680)	0.084	
**Smoke**			0.439						0.658
never	-0.125(-0.248,-0.002)	**0.047**		ref	-0.037(-0.349, 0.276)	-0.197(-0.505, 0.111)	-0.209(-0.467, 0.049)	**0.04**	
former	-0.234(-0.418,-0.050)	**0.016**		ref	-0.373(-0.665,-0.080)	-0.316(-0.674, 0.043)	-0.436(-0.724,-0.147)	**0.016**	
now	-0.12(-0.293, 0.053)	0.159		ref	-0.052(-0.512, 0.408)	-0.077(-0.528, 0.375)	-0.198(-0.586, 0.189)	0.275	

Notes: The LgTFs was converted from a continuous variable to a categorical variable.

Data are presented as OR (95% CI). P value for trend was calculated by entering median values for each LgTFs quartile as a continuous variable in the logistic model. Each stratification adjusted for all factors (age, gender, education level, race/ethnicity, annual family income, PIR, BMI, alcohol consumption, smoking status) except the stratification factor itself. Abbreviations: PIR, poverty index ratio; BMI, body mass index; FT3, free triiodothyronine; TT4, total thyroxine; TT3, total triiodothyronine; LgTFs, Logarithm-transformed total flavonoids; OR, odds ratio; CI, confidence interval; Ref, reference.

## Discussion

For the first time, this cross-sectional cohort study evaluated the associations of LgTFs with thyroid function in 4,949 US participants based on NHANES (from 2007 to 2010). The results confirmed that LgTFs was negatively correlated with TT4. LgTFs was negatively correlated with TT3, FT3 and positively associated with the FT4/TT4 ratio. However, after adjusting for covariates, this correlation became insignificant. Furthermore, the interaction terms suggested that annual family income and PIR might influence the aforementioned associations.And we also found a U-shaped relationship between LgTFs and FT3.

In this study, we provided the total flavonoids intake and the effect of different variables on the dietary flavonoids. As shown in previous study [17], the mean total flavonoids was 344.83 mg/day. Our study was 228.945 mg/day, which is much lower than previous study could be due to excluded proanthocyanidins and used a different version of the flavonoid database. The proanthocyanidin content of foods is excluded because there are not enough data available for food or beverages analyzed [18]. Our study also showed that total flavonoids consumption increased with annual family income, PIR and education level, which is consistent with previous studies [10,19,20].

Other studies have shown that some flavonoids may be beneficial for breast, gastric, lung cancer, thyroid, and liver cancer [21–24]. The finding of Tian-Shin Yeh et al. showed that many flavonoid-rich foods were significantly associated with lower odds of subjective cognitive decline [25]. Although flavonoids have been shown to have some health benefits, they can also cause adverse effects. Moudgal et al. [26] firstly reported that rats fed flavonoids can inhibit thyroid hormone biosynthesis and decrease thyroid iodide uptake, leading to hypothyroidism and goiter. And other researchers showed that some flavonoids were capable of inhibiting thyroid peroxidase activity and iodide organification by different mechanisms, capable of reducing serum T3 and T4 levels and increasing TSH levels, even leading to thyroid gland growth or goiter [27–30]. However, we did not see an increase in TSH levels in our study. The reasons for explaining this discrepancy result is that we only used the average of two days of 24h flavonoids dietary data to investigate the relationship with thyroid function, did not have a long time follow-up. Furthermore, the species and dose of flavonoids used were different in other experiments.

In subgroup analyses stratified by gender, we found differences in the relationship between LgTFs and serum TT4, and the strongest relationship in the female group. Previous data have shown that all thyroid diseases, including thyroid nodules, hypothyroidism, hyperthyroidism, and thyroid cancer, were more common among women than among men and the incidence increased with age [31–34]. These observations suggest that the hypothalamic-pituitary-thyroid (HPT) axis homeostasis of women is more susceptible to environmental, dietary, and endocrine factors [35].

Previous studies have shown that socioeconomic status is an important determinant of differences in food intake, the intake of flavonoids increased with income level [20,36,37]. And in the high annual family income and the high PIR subgroup, we found that the negative association between LgTFs and TT4 became more pronounced. Meanwhile, we also performed subgroup analyses of age, education level, race, BMI, alcohol consumption, and smoking status factors, but there was no significant interaction for these factors.

There are certain limitations to this study. As in other cross-sectional studies, we were unable to collect a long time follow-up data to establish the causal relationship between total flavonoids and thyroid hormone. Second, we excluded participants with cancer, juveniles, or pregnant, which may not apply to all Americans. Finally, environmental and other residual confounders were not taken into account, which could impact the association of total flavonoids intake with thyroid function.

## Conclusion

In summary, our study indicated that a higher intake of total flavonoids in the diet was negatively associated with TT4. Furthermore, the association became more pronounced in adults with high annual family income and high PIR. And we also found a U-shaped relationship between LgTFs and FT3. These findings may provide guidance for diet guidelines with thyroid dysfunction to some extent in the future.
